# Using Chromatin Architecture to Understand the Genetics and Transcriptomics of Juvenile Idiopathic Arthritis

**DOI:** 10.3389/fimmu.2018.02964

**Published:** 2018-12-14

**Authors:** Haeja Kessler, Kaiyu Jiang, James N. Jarvis

**Affiliations:** ^1^Department of Pediatrics, University at Buffalo Jacobs School of Medicine and Biomedical Sciences, Buffalo, NY, United States; ^2^Genetics, Genomics, and Bioinformatics Program, University at Buffalo Jacobs School of Medicine and Biomedical Sciences, Buffalo, NY, United States

**Keywords:** juvenile idiopathic arthritis (JIA), genetics, enhancer, non-coding genome, topically associated domains

## Abstract

The presence of abnormal gene expression signatures is a well-described feature of the oligoarticular and polyarticular forms of juvenile idiopathic arthritis. In this review, we discuss how new insights into genetic risk for JIA and the three dimensional architecture of the genome may be used to develop a better understanding of the mechanisms driving these gene expression patterns.

The completion of the first assembly of the human genome was accompanied by considerable surprise with the discovery that only ~2% of the genome contained protein-encoding genes (1). The lay press seized on these findings and disseminated the idea that 98% of human genomes contained so-called “junk DNA,” on the assumption that the only things of interest in genomes were the protein-coding sequences. Subsequent genome annotation efforts, such as the Encyclopedia of DNA Elements (ENCODE) and Roadmap Epigenomics in the United States and Blueprint Epigenomics in Europe, demonstrated that much of this so-called “junk DNA” actually encoded RNA molecules (2, 3) or contained regulatory regions that played critical roles in fine-tuning transcription during development, cell differentiation, and in response to external stimuli (4).

The era of genome-wide association studies (GWAS), which were undertaken to better understand the genetic contribution to a broad range of complex traits, brought additional surprises. Investigators were perplexed to find that the strongest genetic associations occurred with single nucleotide polymorphisms (SNPs) that were located within non-coding regions of the genome. Indeed, taken together, GWAS SNPs are far more likely to occur in intronic or intergenic regions than in either exons or gene promoters (4).

These findings provide the basis for our inquiry into the causes of the well-described transcriptional abnormalities in JIA. In this paper, we will review the wealth of literature demonstrating abnormal patterns of gene expression in the peripheral blood cells of children with juvenile idiopathic arthritis (JIA). We will then discuss the role of non-coding genomic elements (especially enhancers) in regulating transcription and how an understanding of these mechanisms may allow a deeper understanding of the JIA-associated transcriptional patterns and/or the genetics of JIA.

In this review, we will focus on the oligoarticular and polyarticular, RF-negative subtypes of JIA, as these subtypes appear to represent a continuous spectrum of shared genetic risk (5). In contrast, systemic-onset (6) and RF+ polyarticular disease (7) show unique clinical and genetic features. We should note, however, that many of the broad ideas that we will present in this review are relevant to these other two JIA subtypes, as they are to most complex genetic traits.

## Transcriptional Abnormalities in JIA: Lessons Learned From Gene Expression Profiling

The emergence of technologies to assess transcription on a genome-wide basis was initially met with considerable optimism that these tools could be used to develop clinical biomarkers (8), to classify heterogeneous patient populations more accurately (9, 10), to develop a better understanding of the mechanisms of therapeutic response (11–13) and to better understand disease pathogenesis (14). Different groups have reported a broad range of transcriptional abnormalities in patients with polyarticular and oligoarticular JIA. These transcriptional abnormalities can be observed in whole blood (12), unsorted white blood cells (buffy coats) (15), peripheral blood mononuclear cells (PBMC) (9, 16), neutrophils (17), and CD4+ T cells (18). These studies have generally identified clusters of interferon gamma (IFNg) and tumor necrosis factor alpha (TNFa) regulated genes that display differential expression in children with active polyarticular JIA when they are compared to healthy control children. An IL8 “signature” can also be seen in both neutrophil (19) and whole blood (14) gene expression studies. In general, whole blood expression studies have suggested complex interactions between innate and adaptive immunity in JIA (14, 20). It is curious to note that, even though the oligoarticular and polyarticular rheumatoid factor (RF)-negative subtypes share common genetic risk loci, the largest published study comparing these two phenotypes found that each was distinguishable from the other at the gene expression level (21). Similarly, RF-negative and RF-positive subtypes, which are genetically distinct (7), have remarkably similar gene expression profiles on whole blood microarray analysis. Thus, it is clear that genetic factors are not the sole drivers of the gene expression abnormalities observed in oligoarticular and polyarticular JIA.

The question arises as to whether these transcriptional abnormalities reflect intrinsic defects in gene regulation in the cells of interest, or merely reflect the inflammatory milieu to which these cells are exposed. After all, elevated levels of a broad range of inflammatory mediators can be observed in the serum (22) or plasma (23) of children with JIA. On the other hand, our group has shown that the transcriptional abnormalities observed in JIA neutrophils are accompanied by aberrations in the metabolism of glucose via the hexose monophosphate shunt (19). Similarly, Throm and colleagues have shown distinct aberrations in interferon gamma-mediated (IFNg) signaling pathways in JIA T cells (24) studied *in vitro*. Finally, neither PBMC nor neutrophil signatures “normalize” after children have achieved clinical remission on medication (CRM), although it's curious to note that the neutrophil aberrations are more prominent (13). We should also note that the intrinsic defect vs. externally-driven hypotheses to explain the distinct transcriptional profiles of JIA peripheral blood cells are not mutually exclusive. Although we are coming to understand the strong effect that the environment (broadly considered) has on peripheral blood gene expression (25), underlying genetics and the immediate external milieu to which the cells are exposed may both play a role in the transcriptional patterns observed in peripheral blood leukocytes of children with JIA.

We were naturally led us to ask whether the emerging knowledge of the genetics of JIA might provide a useful framework from which to understand the mechanisms driving the transcriptional abnormalities in JIA peripheral blood cells. This necessarily leads to a brief discussion of the genetics on JIA. This discussion will not be comprehensive, and the reader wishing to have a deeper understanding is invited to read the recent reviews available on this subject (26, 27).

## GWAS and the Genetics of JIA

Using candidate gene approaches, GWAS, and genetic fine mapping studies, investigators have identified >30 genetic loci associated with JIA (26–28). Each of these 3 approaches queries a single or small groups of SNPs (candidate gene approaches) or large numbers of SNPs (GWAS and genetic fine mapping studies) and asks whether specific alleles occur more frequently in individuals with a specific disease or phenotype than they do in controls (29) to identify alleles that have a strong *association* with the disease or trait of interest. It is important to note, however, that SNPs identified by such studies may not be the actual genetic variants that exert the biological effects that confer risk. This is because the so-called tag SNPs (or candidate SNPs used in candidate gene approaches) are in linkage disequilibrium (LD) with hundreds or thousands of other SNPs in the same region, any one of which may exert risk-enhancing biological effects. To use an analogy, GWAS can be understood as something like a crude global positioning satellite (GPS) that can tell you, say, that you are on the M1, somewhere between London and Sheffield, assuring you that you are not in Cornwall, but not providing any information as to whether you're actually closer to Leicester or Nottingham. Thus, GWAS have merely identified regions of the genome where genetic risk may be exerted. These regions can be referred to as “LD blocks” or, more commonly, risk haplotypes. It is common to refer to the risk haplotypes in JIA (and other complex traits) by the gene nearest to the tag SNP, and this has led to the common misunderstanding that the GWAS SNP: (1) is the one that actually exerts the relevant biological effects and (2) exerts those effects on the nearest gene. Neither is necessarily the case.

**Figure 1:**
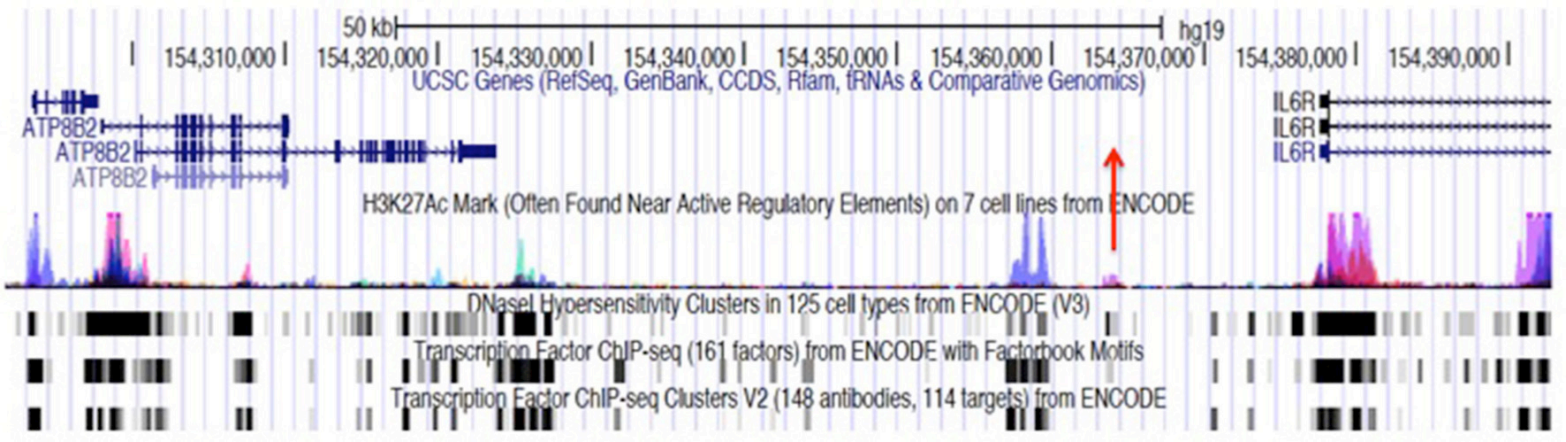
Genome browser screen shot showing the JIA-associated *IL6R* locus, identified by the tag SNP, rs11265608, and encompassing the region chr1:154291718–154379369. Transcription is from left to right. This locus consists largely of an intergenic region between the *IL6R* and *ATP8B2* genes. The haplotype block also encompasses the first exon and intron of the *IL6R* gene and most of the coding sequence of the *ATP8B2* gene. The position of the tag SNP, rs11265608, is indicated by the red arrow. ChIPseq peaks for the histone mark H3K27ac from ENCODE and Roadmap Epigenomics data, are shown with the blue/magenta peaks. Transcription factor (TF) binding data and DNAse hypersensitivity data (also from ENCODE and Roadmap Epigenomics) are is represented black and gray boxes at the bottom of the figure. The regions where TF and H3K27ac marks overlap are putative enhancers. Almost identical chromatin architecture is seen in both CD4+ T cells and neutrophils within this haplotype. Note that neutrophils also express an intergenic RNA molecule at chr1:154350688-154350783 (not shown).

Figure 1 illustrates this point. *IL6R* is a haplotype that was identified on the genetic fine mapping study published by Hinks et al. (5). The haplotype spans the region marked by the genomic coordinates, chr1:154291718-154392674, a length of >100,000 bp. The reader will also note that there are functional elements *other than* the coding genes (*ATP8B2* and a portion of *IL6R*); this region is also characterized by prominent, overlapping H3K4me1/H3K27ac histone marks (for simplicity, only the H3K27 marks are shown in the figure), and dense transcription factor binding even in non-coding regions (i.e., within introns and the intergenic area). These chromatin features are commonly associated with enhancers, about which it is useful to say more as we query the possible role of genetics in driving the transcriptional abnormalities observed in JIA.

## JIA and Enhancers

H3K4me1 and H3K27ac refer to covalent modifications to the tails of histones, epigenetic features that typically accompany enhancers, as noted above. One of the striking features that emerged from multiple GWAS studies was the high frequency with which the genetic “hits” occurred within H3K4me1/H3K27ac-marked regions of pathologically-relevant cells for the traits of interest (4). The reverse is also true: if one maps enhancer elements in specific cell types, those mapped regions are highly enriched in GWAS-identified SNPs (30) for diseases that affect those cells/tissues. We have reported that the risk loci for both JIA (31, 32) and systemic lupus (33), are highly enriched (compared to randomly-selected regions of functional chromatin) for H3K4me1/H3K27ac histone marks. These findings have led us to suggest that much of the genetic risk for JIA is exerted through altered function of these enhancers.

**Figure 2:**
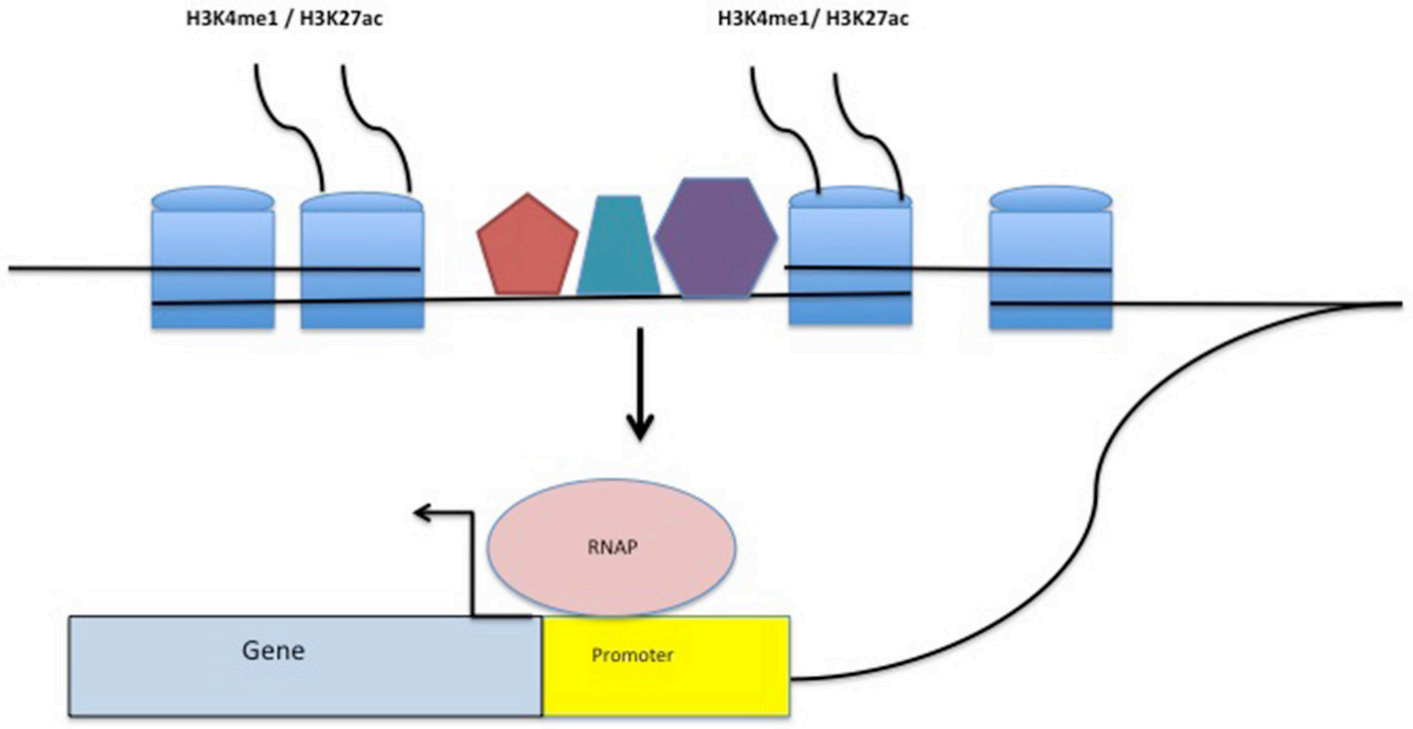
Structure of a typical enhancer. H3K4me1/H3K27ac-marked histones flank a region densely bound by transcription factors (TFs-represented by the pentagon, hexagon, and trapezoid). The enhancer complex physically interacts with gene promoters, stabilizing TFs within the promoter as well as RNA polymerase (RNAP) binding, facilitating transcription.

**Figure 3:**
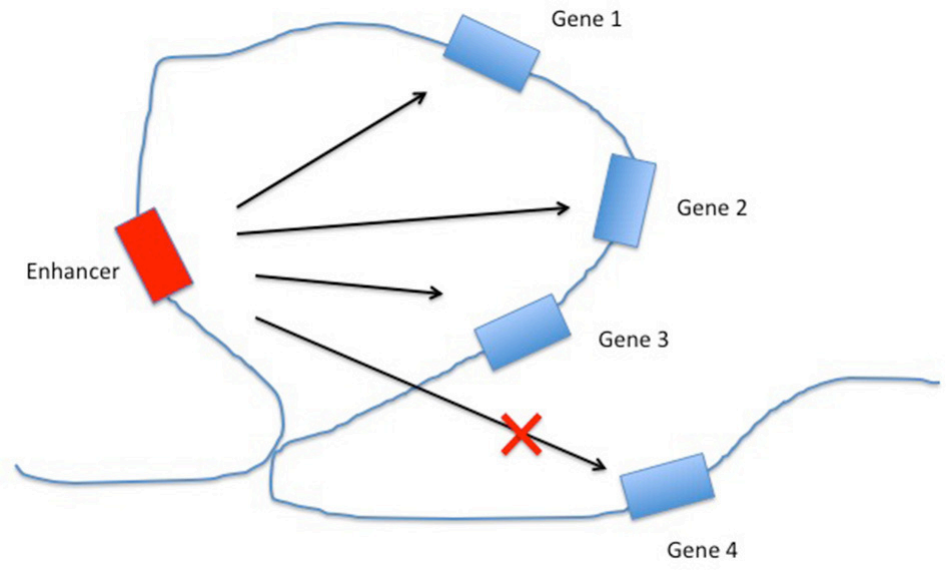
Regulation of gene expression by enhancers within topologically associated domains (TADs). The chromatin loop structure facilitates interactions between the enhancer and some (or all) of the genes within the same TAD, while constricting the interactions between the enhancer and genes outside the TAD.

**Figure 4:**
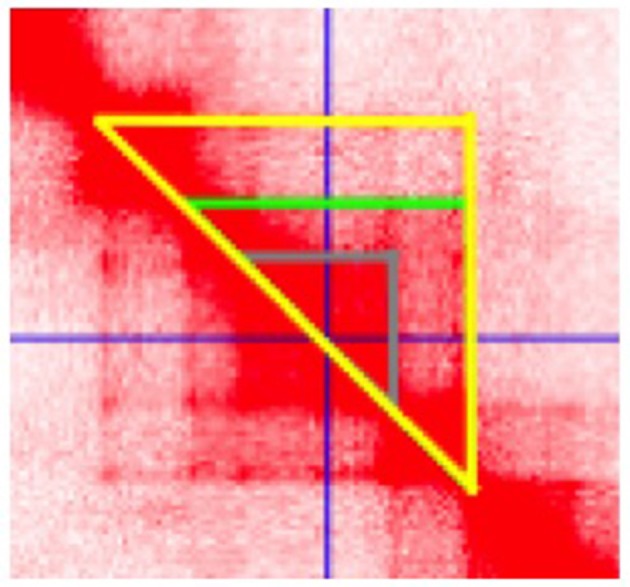
Juicebox software visualization of the topologically associated domain (TAD) containing the JIA-associated *IL6R* haplotype. Data are from HiC analysis of K562 cells. The cursor (crossed blue lines) is set at the center of a putative enhancer in the region spanning chr1:154, 353, 745–154, 361, 534. Three distinct chromatin loops are identified, as indicted by the gray, green, and yellow triangles, suggesting complex chromatin architecture in this region.

Enhancers are non-coding DNA elements that play an important role in regulating gene expression, serving as rheostats that adjust gene expression to fit finely-tuned physiologic contexts (34). Enhancers have a characteristic structure that includes the presence of open chromatin bound by multiple transcription factors (TFs) flanked by H3K4me1/H3K27ac-marked boundaries (Figure 2). These TFs form a complex that typically includes p300, mediator, and cohesin, which together facilitate looping and physical contact with the promoters of target genes. While many enhancers function constitutively, others remain latent, activated only by specific cellular or environmental triggers (35). It is important, from the standpoint of our understanding the genetics and pathobiology of JIA, to note that enhancers do not always regulate the nearest gene. Furthermore, a given enhancer may regulate more than one gene, and a given gene may be regulated by more than one enhancer. Although enhancers may not regulate the nearest gene, they typically regulate genes within the same chromatin loop or *topologically associated domain* (TAD). Our understanding of the three-dimensional structure of chromatin within the nucleus of eukaryotic cells has expanded significantly in the past 10 years, and readers interested in learning more on this fascinating topic and its relation to human disease may wish to consult some of the recent reviews (36–39). For the purposes of this review, TADs can be considered the basic chromatin loop structure that regulates enhancer-promoter interactions, as shown in Figure 3. TADs can be identified using non-targeted chromatin conformation techniques such as HiC (40), and visualized using publically available software such as JuiceBox (41). Figure 4 shows how the TAD for the IL6R locus can be visualized. The larger loop contains multiple genes, including *IL6R, IL6R-AS1, ATP8B2, SHE, TDRD10, UBE2Q1*, and *UBE2Q1-AS1*. Any or all of these genes may be regulated by enhancer(s) within the *IL6R* locus, and thus *dys*regulated by genetic variants that disrupt or alter enhancer function within this locus.

## Querying the Risk Haplotypes for Differentially Expressed Genes

Now we return to the question of the role of genes vs. environment (broadly considered) in driving the gene expression abnormalities in JIA. We recently reported on results of whole blood expression studies from children enrolled in the NIH-funded Trial of Early Aggressive Therapy in JIA (TREAT trial) (14). In that study, we identified 158 genes that showed differential expression when we compared children with new-onset polyarticular JIA with healthy controls. We then used conventional computational techniques to intersect the differentially expressed genes with the known JIA haplotypes. We were unable to identify a single differentially expressed gene within the known JIA haplotypes. We have subsequently repeated this technique using all publically available gene expression data from children with oligoarticular and polyarticular JIA and gotten the same result. This has led us to the conclusion that *if* genetic variants associated with JIA influence the observed transcriptional patterns, they must either do so within specific leukocyte subsets that are not detectable using whole blood, or they must act on longer-range chromatin interactions. Once again these are not mutually exclusive considerations. We anticipate that the emergence of single-cell technologies and perhaps the development of disease-specific three-dimensional chromatin maps will allow us to understand the genetic contribution (if any) to the observed peripheral blood expression abnormalities.

## Bringing It All Back Home: A Broader Look at the JIA Risk Loci

Let us return to Figure 1, which shows the multiple features within the *IL6R* haplotype, which occupies the genomic coordinates chr1:154291718-154392674 and spans >100,000 bp. This risk locus is representative of the other JIA risk loci, which almost invariably show the same of similar chromatin features (31, 32).

The reader will note immediately that the haplotype contains only a portion of the *IL6R* gene as well as an additional gene, *ATP8B2*. The protein product of *ATP8B2* is an ATPase, and the gene is expressed in both lymphoid cells as well as in myeloid cells such as macrophages (42); its exact role in these cells has not been investigated in any detail, although it is reasonable to speculate that it is involved in cellular energy production and utilization. Using RNA sequencing in human neutrophils, we have also shown that the haplotype also contains at least one non-coding intergenic RNA (ncRNA), a species of RNAs that are important in regulating both three dimensional chromatin architecture (43) and gene expression (44). We have previously shown that the presence of ncRNA molecules expressed in neutrophils is a common feature within the JIA risk loci (31, 32) and the presence of such RNA species in pathologically relevant cells is a characteristic that the JIA risk loci share with those of most other complex traits.

Finally, we note once again the rich in H3K4me1/H3K27ac histone marks, which overlap with abundant transcription factor binding sites, features that can be observed particularly in CD4+ T cells (ENCODE and Roadmap Epigenomics data) as well as our own neutrophil ChIP-seq data (31). Indeed the chromatin architecture at this locus suggests the presence of multiple intronic and intergenic enhancers in both cell types.

These observations raise multiple questions regarding the mechanism(s) through which genetic variants within this locus impinge on immune function. Do genetic variants alter the structure of the ncRNA or its regulation? Do they alter the expression of *ATP8B2 or IL6R* through alterations in their promoters? Do they alter the function of one or more of the enhancers? If so, what genes are dysregulated by altered enhancer function? If genetically-mediated dysregulation isn't exerted on peripheral blood cells, then where is it exerted?

These aren't either/or questions. That is, different genetic variants in different individuals might alter one or more of the genomic functions within the JIA haplotypes to different degrees. This might explain, for example, the considerable differences we see between individuals with JIA at both the phenotypic and gene expression levels. Indeed, we believe that one of the reasons why there is so much overlap in the genetic associations seen for a broad range of autoimmune/inflammatory diseases in the fact that these loci contain *multiple* important genomic elements which, if perturbed, could lead to an immune phenotype.

## Where Do We Go From Here?

We are still rather in the dark as to the origin of the abnormal transcriptional signatures in JIA. It's clearly not as straightforward as we initially thought it might be: that polymorphisms in gene promoters, for example, would lead to alterations in gene expression that might be easily observed in cells or in serum protein levels. It should also be sufficiently clear to the reader that there is limited, if any, utility in trying to understand genetic mechanisms in JIA by focusing solely on the coding functions of the genes in close proximity to the SNPs identified on GWAS and genetic fine mapping studies. This “nearest gene” focus ignores the broader chromatin architecture in which the biologically relevant variants are likely to operate. We propose that future studies of individual JIA-associated loci consider not only the entire risk haplotype and the multiple genomic elements contained within it, but also broaden the inquiry to include three dimensional chromatin architecture and the genes included within the TADs that incorporate the risk haplotypes. This means that there is going to be a lot of work to do at each risk locus.

Enhancers seem to be the logical place to start, given their demonstrated importance in JIA (31, 32) and rheumatic diseases in general (45, 46). While the chromatin signatures within the JIA risk loci are strong indicators that these regions have enhancer function, the specific functional regions will need to be identified and verified empirically using reporter assays. Once the specific functional regions are identified, it will be a straightforward task to clarify the effects of genetic variants within these functional regions. Publically available data like the 1000 Genomes Project will give investigators a large but finite number of common genetic variants (allele frequencies >1%) to test within the defined regions. Our recent whole genome sequencing data from children with polyarticular, RF-negative JIA (47) have been made available to investigators through the National Center for Biotechnology Information (https://www.ncbi.nlm.nih.gov/bioproject/?term=PRJNA343545) and will provide the scientific community with a rich list of rare variants to interrogate.

Once enhancer function is confirmed and the biologically-relevant genetic variants identified, we still have before us the task of identifying the genes (dys)regulated by the enhancers on which genetic variance operates. To accomplish this aim, our laboratory is taking advantage of the fact that most enhancers regulate genes within the same TAD. We are therefore using an epigenome editing approach (48) to attenuate enhancer function and identify genes whose expression is altered when specific enhancers are attenuated.

## Conclusion

The identification of aberrant transcriptional patterns in the peripheral blood cells of children with JIA has opened the door to intriguing inquiries into the epigenetics and genetics of this family of diseases. With regard to the latter, our growing understanding of the structure and function of mammalian genomes makes it imperative that we broaden our investigations beyond the “nearest gene” to GWAS tag SNPs. Rather, developing a mechanistic understanding of how and where genetic variants alter transcription and immune function will require a complete understanding of the range of genomic functions altered by variants within the risk haplotypes. Furthermore, the field will require a detailed understanding of the larger chromatin “neighborhoods” within which each of the haplotypes resides. These are reachable goals that are most likely to be achieved by a focused and coordinated effort among the different pediatric rheumatology genetics research consortia to prioritize loci, share expertise and reagents, and develop plans for using genetic information to inform clinical care.

## Author Contributions

JJ developed the concept for the paper and assisted in the writing. HK assisted in the 3D chromatin analysis. KJ assisted in developing the concepts and generated data cited in this paper.

### Conflict of Interest Statement

The authors declare that the research was conducted in the absence of any commercial or financial relationships that could be construed as a potential conflict of interest.
